# Supplementary material for: ABC Transporter C1 Prevents Dimethyl Fumarate from Targeting Alzheimer’s Disease
Source: Biology (Basel). 2023 Jun 29;12(7):932. doi: 10.3390/biology12070932 (PMC10376064; doi:10.3390/biology12070932)
Supplement: Supplementary file 1 [file biology-12-00932-s001.zip › biology-2451083-supplementary.pdf]

Supplementary Materials – Table S1 – Dimethyl fumarate tested in animal models of 'Alzheimer's disease'

Abbreviations: AD – Alzheimer's disease; DMF - dimethyl fumarate

Publication	Animal model	Sex and age	DMF application duration and dosage	Main findings, readout
**Transgene mouse models**				
Rojo et al. (2018) [1]	APP^{V717I} / TAU^{P301L} transgenic mice (Nrf2-WT group) (C57BL/6J background)	both, 9 months old	DMF for 42 days; 100 mg/kg/every 2 days by oral gavage	The study investigated the role of a transcriptional factor Nrf2 in a mouse model of AD with both tauopathy and amyloidopathy. The researchers found that mice deficient in Nrf2 (Nrf2-KO) had more severe inflammatory changes in the brain compared to wild-type mice (Nrf2-WT), as evidenced by increased levels of pro-inflammatory cytokines and microglial activation. Nrf2-deficiency was also associated with greater accumulation of tau and Aβ in the brain, as well as increased oxidative stress and neuronal damage. Overall, the findings suggested that Nrf2 plays an important role in modulating inflammatory and oxidative stress responses in AD, and that deficiency in this transcription factor may exacerbate the pathological processes underlying the disease.<br>The study highlights the potential of Nrf2 as a therapeutic target for AD, and suggests that drugs that activate Nrf2 signaling pathways may have potential as disease-modifying treatments. DMF treatment reduced motor alteration and improved memory in the Nrf2-WT group with APP and TAU expression (*Thy1*-promoter).

Mohle et al. (2021) [2]	APPPS1-21 transgenic mice (C57BL/6J)	females, 40d or 60d of age	DMF for 40 days; 75 mg/kg daily oral gavage	This study aimed to investigate the effects of DMF on cognitive decline and β-amyloidosis in a mouse model of AD called APPPS1-21.<br>The researchers found that DMF treatment **did not improve cognitive performance** in the APPPS1-21 mice, as assessed by a range of tests including the Morris water maze and the novel object recognition task.<br>DMF treatment was also found to have **no significant effect** on the accumulation of β-amyloid plaques in the brains of the APPPS1-21 mice, as assessed by immunohistochemistry and ELISA assays.<br>The study suggests that **DMF may not be an effective therapeutic intervention for AD** in this mouse model, at least in female mice, and that further studies are needed to fully understand the potential of DMF in treating the disease.<br>The findings also highlight the importance of considering sex differences in preclinical studies of AD, as previous studies have shown that female mice may exhibit different responses to therapeutic interventions compared to males.

Publication	Animal model	Sex and age	DMF application duration and dosage	Main findings, readout
**Induction-of-pathology models** (rats and mice)<br>*These models are **not** AD or AD-like models!*				

Majkutewicz *et al.* (2016) [3]	Wistar rats; Streptozotocin-induced; i.c.v. delivery	Males, 4 months old	DMF for 21 days; 0.4% DMF in the rat chow by body weight	The study investigated the effects of DMF on rats that had been given intracerebroventricular streptozotocin to induce spatial memory impairment and hippocampal neurodegeneration. Treatment with DMF was found to reduce the severity of spatial memory impairment and neurodegeneration in the rats. Rats that received DMF performed better on a spatial memory task than those that did not receive DMF. DMF treatment also resulted in less degeneration in the hippocampus, a brain region important for memory and learning. These findings suggest that DMF may have potential as a neuroprotective agent for the treatment of memory impairment and neurodegenerative disorders.
Majkutewicz et al. (2018) [4]	Wistar rats; Streptozotocin-induced; i.c.v. delivery	Males, 22 months old	DMF for 21 days; 0.4% DMF in the rat chow by body weight	The study aimed to investigate the effects of DMF on cognitive and neuropathological features in a rat model induced by streptozotocin.<br>The researchers found that DMF treatment improved cognitive performance in the STZ-induced rats, as assessed by a range of tests including the Morris water maze and the novel object recognition task.<br>DMF treatment was also found to reduce neuropathological features of AD in rat brains, including amyloid-beta (Aβ) accumulation, neuroinflammation, and oxidative stress.<br>Interestingly, the effects of DMF treatment were found to be age-dependent: the treatment was most effective in younger rats (4-5 months old), while in older rats (8-9 months old) the effects were less pronounced.<br>Overall, the findings suggest that DMF may have potential as a therapeutic intervention for AD, particularly in the early stages of the disease. However, the age-dependent effects of the treatment should be taken into account in future studies and in the development of DMF-based therapies for AD.
Wrona et al. (2022) [5]	Wistar rats; Streptozotocin-induced; i.c.v. delivery	Males, 4 or 22 months old	DMF for 21 days; 0.4% DMF in the rat	The study investigated the effects of DMF on neuroinflammation and cognitive function in aged rats following induction of neuroinflammation with streptozotocin.

			chow by body weight	The researchers found that DMF treatment reduced the expression of inflammatory markers in the peripheral blood, including interleukin-1β (IL-1β), interleukin-6 (IL-6), and tumor necrosis factor-α (TNF-α).<br>DMF treatment was also found to reduce the activation of microglia and astrocytes in the brain, as well as reducing oxidative stress and apoptosis.<br>The study further showed that the protective effects of DMF were associated with improved cognitive performance in the rats, as assessed by a range of behavioral tests including the Morris water maze and the passive avoidance test.<br>The findings suggest that DMF may have therapeutic potential for preventing neuroinflammation and cognitive decline in aged individuals, and highlight the importance of investigating the mechanisms underlying the effects of DMF in preclinical models of neuroinflammation.<br>However, further studies are needed to confirm the potential of DMF as a treatment for neuroinflammatory conditions in humans, including exploring the optimal dose and treatment duration.
Abd El-Fatah et al. (2021) [6]	Wistar rats; Induction using D-galactose (D-Gal) administered to ovariectomized (OVX) rats to induce the AD-like model	Females, 18 months old	DMF for 56 days;<br>45 mg/kg by oral administration	The study investigated the effects of DMF on cognitive impairment and pathological features of 'AD-like' rat model, which was induced by D-galactose and ovariectomy.<br>The researchers found that DMF treatment improved cognitive performance in the rats, as assessed by a range of behavioral tests including the Morris water maze and the novel object recognition task.<br>DMF treatment was also found to reduce the accumulation of tau and Aβ in the brain, as well as reducing oxidative stress and inflammation.<br>The study further showed that the protective effects of DMF were associated with the modulation of several cellular signaling pathways, including the AMPK/SIRT-1, AKT/CREB/BDNF, AKT/GSK-3β, adiponectin/Adipo1R, and NF-κB/IL-1β/ROS pathways.<br>The findings suggest that DMF may have therapeutic potential for AD by targeting multiple pathological processes, and highlight the importance of

				investigating the mechanisms underlying the effects of DMF in preclinical models of the disease. However, further studies are needed to confirm the potential of DMF as a potential treatment for AD patients.
Sun et al. (2022) [7]	C57BL/6J mice; Induction of AD-like model by combined injection of Aβ_{1-42} peptide and ibotenic acid into the hippocampus	Males, 8 weeks old	DMF for 21 days; 48 mg/kg by intragastric administration	The study investigated the effects of DMF on cognitive impairment and pathological features of AD in a mouse model induced by intrahippocampal injections of Aβ_{1-42} and ibotenic acid. The researchers found that DMF treatment improved cognitive performance in the AD mice, as assessed by a range of behavioral tests including the Morris water maze. DMF treatment was also found to inhibit the mitochondrial dysfunction and to reduce Aβ deposition, as well as reducing oxidative stress and inflammation. The study further showed that the protective effects of DMF were associated with modulation of several cellular signaling pathways, including the Nrf2/HO-1 and NF-κB pathways. The findings suggest that DMF may have therapeutic potential for AD by targeting multiple pathological processes, and highlight the importance of investigating the mechanisms underlying the effects of DMF in preclinical models of the disease. However, further studies are needed to confirm the potential of DMF as a treatment for AD in humans, including exploring the optimal dose and treatment duration.

References

1. Rojo, A. I.; Pajares, M.; Garcia-Yague, A. J.; Buendia, I.; Van Leuven, F.; Yamamoto, M.; Lopez, M. G.; Cuadrado, A., Deficiency in the transcription factor NRF2 worsens inflammatory parameters in a mouse model with combined tauopathy and amyloidopathy. *Redox Biol* **2018,** 18, 173-180.

2. Mohle, L.; Brackhan, M.; Bascunana, P.; Pahnke, J., Dimethyl fumarate does not mitigate cognitive decline and beta-amyloidosis in female APPPS1 mice. *Brain Res* **2021,** 1768, 147579.

3. Majkutewicz, I.; Kurowska, E.; Podlacha, M.; Myslinska, D.; Grembecka, B.; Rucinski, J.; Plucinska, K.; Jerzemowska, G.; Wrona, D., Dimethyl fumarate attenuates intracerebroventricular streptozotocin-induced spatial memory impairment and hippocampal neurodegeneration in rats. *Behavioural brain research* **2016,** 308, 24-37.

4. Majkutewicz, I.; Kurowska, E.; Podlacha, M.; Myslinska, D.; Grembecka, B.; Rucinski, J.; Pierzynowska, K.; Wrona, D., Age-dependent effects of dimethyl fumarate on cognitive and neuropathological features in the streptozotocin-induced rat model of Alzheimer's disease. *Brain Res* **2018,** 1686, 19-33.

5. Wrona, D.; Majkutewicz, I.; Swiatek, G.; Dunacka, J.; Grembecka, B.; Glac, W., Dimethyl Fumarate as the Peripheral Blood Inflammatory Mediators Inhibitor in Prevention of Streptozotocin-Induced Neuroinflammation in Aged Rats. *J Inflamm Res* **2022,** 15, 33-52.

6. Abd El-Fatah, I. M.; Abdelrazek, H. M. A.; Ibrahim, S. M.; Abdallah, D. M.; El-Abhar, H. S., Dimethyl fumarate abridged tauo-/amyloidopathy in a D-Galactose/ovariectomy-induced Alzheimer's-like disease: Modulation of AMPK/SIRT-1, AKT/CREB/BDNF, AKT/GSK-3beta, adiponectin/Adipo1R, and NF-kappaB/IL-1beta/ROS trajectories. *Neurochem Int* **2021,** 148, 105082.

7. Sun, X.; Suo, X.; Xia, X.; Yu, C.; Dou, Y., Dimethyl Fumarate is a Potential Therapeutic Option for Alzheimer's Disease. *J Alzheimers Dis* **2022,** 85, (1), 443-456.
